# Supplementary material for: Novel Driver Strength Index highlights important cancer genes in TCGA PanCanAtlas patients
Source: PeerJ. 2022 Aug 11;10:e13860. doi: 10.7717/peerj.13860 (PMC9375969; doi:10.7717/peerj.13860)
Supplement: Supplemental Information 2 [file peerj-10-13860-s002.zip › COHORTS/genes plots/age/2021_8_16_14_9_distribution_cohorts_55-59.pdf]

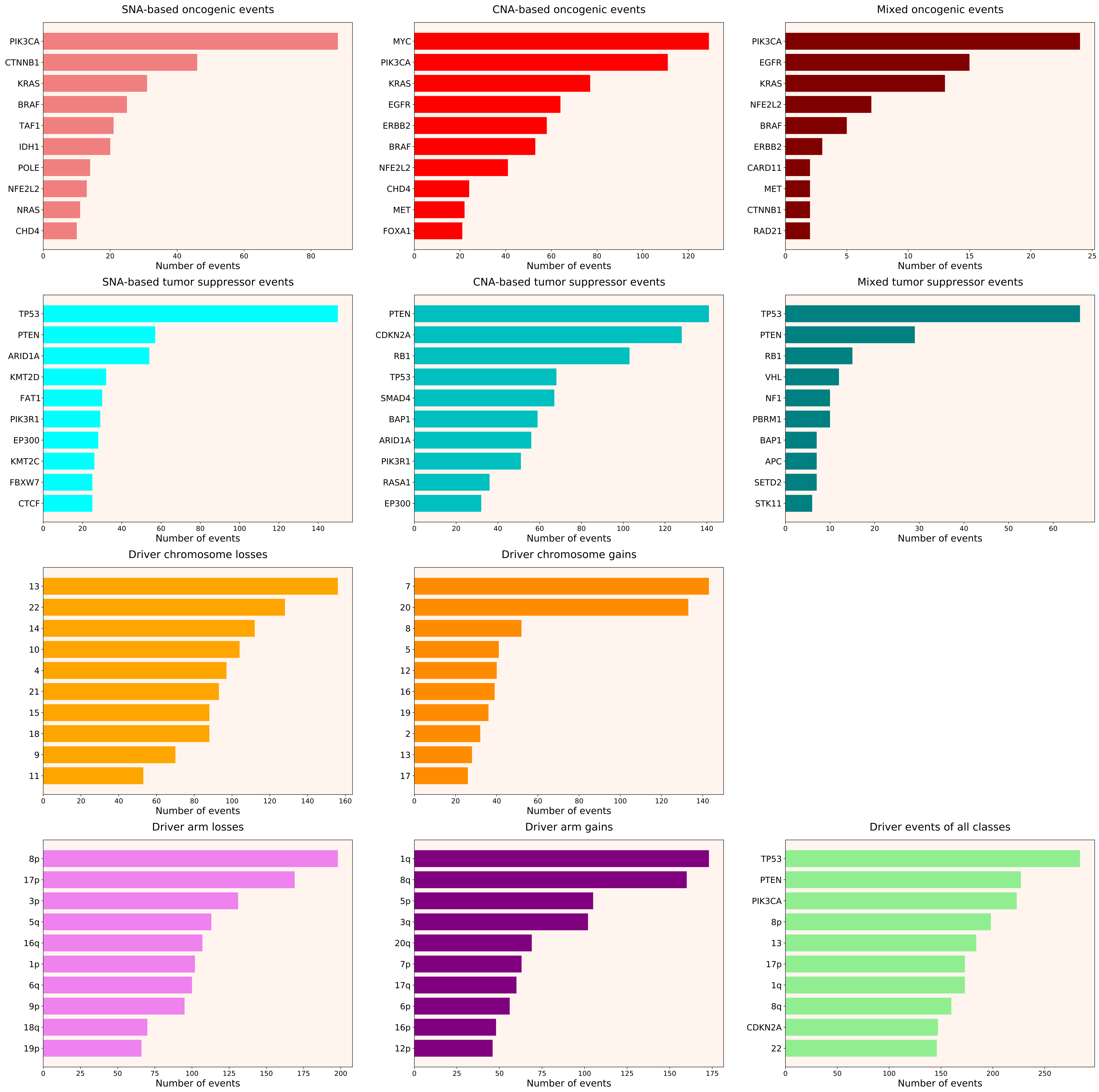
SNA-based oncogenic events
PIK3CA
CTNNB1
KRAS
BRAF
TAF1
IDH1
POLE
NFE2L2
NRAS
CHD4
Number of events
CNA-based oncogenic events
MYC
PIK3CA
KRAS
EGFR
ERBB2
BRAF
NFE2L2
CHD4
MET
FOXA1
Number of events
Mixed oncogenic events
PIK3CA
EGFR
KRAS
NFE2L2
BRAF
ERBB2
CARD11
MET
CTNNB1
RAD21
Number of events
SNA-based tumor suppressor events
TP53
PTEN
ARID1A
KMT2D
FAT1
PIK3R1
EP300
KMT2C
FBXW7
CTCF
Number of events
CNA-based tumor suppressor events
PTEN
CDKN2A
RB1
TP53
SMAD4
BAP1
ARID1A
PIK3R1
RASA1
EP300
Number of events
Mixed tumor suppressor events
TP53
PTEN
RB1
VHL
NF1
PBRM1
BAP1
APC
SETD2
STK11
Number of events
Driver chromosome losses
13
22
14
10
4
21
15
18
9
11
Number of events
Driver chromosome gains
7
20
8
5
12
16
19
2
13
17
Number of events
Driver arm losses
8p
17p
3p
5q
16q
1p
6q
9p
18q
19p
Number of events
Driver arm gains
1q
8q
5p
3q
20q
7p
17q
6p
16p
12p
Number of events
Driver events of all classes
TP53
PTEN
PIK3CA
8p
13
17p
1q
8q
CDKN2A
22
Number of events
